# The characteristics of residents with unawareness of hepatitis C virus infection in community

**DOI:** 10.1371/journal.pone.0193251

**Published:** 2018-02-22

**Authors:** Pin-Nan Cheng, Yen-Cheng Chiu, Hung-Chih Chiu, Shih-Chieh Chien

**Affiliations:** Division of Gastroenterology and Hepatology, Department of Internal Medicine, National Cheng Kung University Hospital, College of Medicine, National Cheng Kung University, Tainan, Taiwan; Centers for Disease Control and Prevention, UNITED STATES

## Abstract

**Background:**

Control of hepatitis C virus infection (HCV) is an increasingly important issue. Enhancing screening coverage is necessary to discover more HCV infected subjects in community. However, a substantial population is unaware of HCV infection that needs more attention.

**Aim:**

The aims of this study were to evaluate the status of HCV infected residents in remote villages, to compare characteristics between already known and unaware HCV infection subjects, and to analyze the disease insights.

**Patients and methods:**

Screening intervention for liver diseases was conducted in remote villages of Tainan City of southern Taiwan from August 2014 to July 2016. Items of screening examinations included questionnaire, blood sampling for liver tests and viral hepatitis markers (hepatitis B surface antigen and anti-HCV antibody), abdominal sonography survey, and liver stiffness measurement by transient elastography. Quantitation of HCV RNA was measured for residents with positive anti-HCV antibody.

**Results:**

A total of 194 (13.5%) out of 1439 participants showed positive for anti-HCV antibody. HCV viremia was detected in 119 (61.3%) residents. Previously unaware HCV infection by questionnaire record was present in 68 (35.1%) of ant-HCV positive residents. By multivariate logistic analysis, unaware HCV infected residents exhibited significantly mild liver fibrosis (OR 0.876, 95% CI 0.782~0.981, p = 0.022), more prevalent of heart diseases (OR 6.082, 95% CI 1.963~18.839, p = 0.002), and less cluster of family history of liver diseases (OR 0.291, 95% CI 0.113~0.750, p = 0.011) when comparing with already known HCV infected residents. Among the 126 already know HCV infected residents, only 59 (46.8%) received antiviral treatment or regular follow-up. No concept or no willing to receive medical care was observed in 44 (34.9%) residents.

**Conclusion:**

In HCV endemic villages of Taiwan, residents with unaware HCV infection comprised about one third of HCV infected residents and exhibited obscure characteristics to identify. Less than half of already known HCV infected residents received adequate medical care. To eliminate HCV infection, vigorous efforts on enhancing screening coverage, educating update knowledge of liver diseases, and linking to medical care are urgently needed.

## Introduction

There are about 71 million people with chronic hepatitis C virus infection world-wide [1–2]. Global control of hepatitis C virus (HCV) infection nowadays is an important healthy issue. As disease progresses, chronic hepatitis C (CHC) can result in the development of liver cirrhosis, hepatocellular carcinoma, and complications of liver diseases [3–5]. CHC also associates with extrahepatic disorders such as cardiovascular events or chronic kidney diseases [6]. Lines of evidence show that successful treatment to clear HCV exerts long-term beneficial effects on either hepatic or extrahepatic outcomes [7,8].

With the advent of direct acting antiviral agents (DAA) in recent years, this new treatment provides patients and physicians an easier, shorter, safer, and highly efficient modality to clear HCV [9,10]. For achieving the goal of HCV elimination set by World Health Organization (reducing new infections by 90% and mortality by 65%) by 2030 [11], efforts to enhance capacity or coverage of screening, to prevent new infection and reduce transmission, and to link infected subjects to medical care and curative treatment needs to be aggressively implemented. Among these issues or efforts, the base of HCV elimination is to expand the coverage of screening and to discover subjects who have HCV infection but not be tested or aware. However, little is known about the features of those subjects who are not aware of HCV infection. Understanding the characteristics of unaware HCV infected subjects and the difference from already known HCV infected subjects is important and may provide highlights to design further massive screening intervention.

In this study, we aimed to analyze the baseline demography of HCV infected residents in community who were already known infection or newly diagnosed infection by screening intervention. Through comparisons between these two kinds of infected residents, features of unaware HCV infected subjects are likely to be explored and recognized. In addition, we also evaluated the insights of liver diseases in already known HCV infected subjects in community to investigate the altitude and knowledge facing HCV infection.

## Materials and methods

### 1. Screening intervention

We conducted screening activities of liver diseases in four remote villages (Jian-Jun, Chi-Koo, Hsin-Hwa, and Shen-Hwa) of Tainan City of southern Taiwan from August 2014 to July 2106. The study was approved by the Institutional Review Board of National Cheng Kung University Hospital. All residents participated in screening activities voluntarily and signed informed written consent. The process of screening intervention was described previously [12]. All patients were fasted for overnight before commencing screening intervention. Briefly, the intervention composed of two parts. The first part included recording baseline characteristics of participants, questionnaire (supporting information), waist/hip circumference measurement, blood sampling for viral markers and liver biochemical tests, abdominal sonography, and liver stiffness measurement by transient elastography (Echosens, France). The reliable measurement of liver stiffness measurement was defined as those valid measurements with less than 30% of Inter-quartile range/median. In questionnaire, current co-morbid diseases, knowledge of chronic liver diseases, detail history of alcohol and viral hepatitis, insights of liver diseases including current or history treatment or follow-up of viral hepatitis, history of occurrence of complications of liver diseases, and family history of liver diseases were asked and recorded by trained nurses. The trained nurses were unaware of HCV infection history of any participated residents. For the awareness of HCV infection, history of HCV examination was clarified and then classified as known HCV infection or unaware HCV infection according to the responded answer. Fatty liver on sonography was defined as the presence of liver-renal echo contrast and bright liver [13]. Those residents who showed fatty liver on sonography were considered to have non-alcoholic fatty liver disease (NAFLD).

The second part held two weeks later after first part. In this time period between first and second part, quantitation of HCV RNA was performed for all residents with positive anti-hepatitis C Antibody (anti-HCV). In the second part, we explained all of the examination reports to participants and referred those who required further evaluation or treatment to our hospital.

### 2. Laboratory tests

Aspartate aminotransferase (AST, upper limit of normal is 38 U/L), alanine aminotransferase (ALT, upper limit of normal is 40 U/L), hepatitis B surface antigen (HBsAg; Architect HBsAg QT assay, Abbott, Chicago, IL), Anti-HCV (ARCHITECT Anti-HCV, ABBOTT, Diagnostics Division, Germany) were tested. Quantitation of HCV RNA (Abbott RealTime HCV quantitative assay; Abbott Molecular Inc., Des Plaines, IL, USA) was further determined in residents with positive anti-HCV.

### 3. Statistical analysis

Data were expressed as mean plus standard deviation. Groups were compared for distributed data by Student’s t-test and for category data by Chi-square test or Fisher’s exact test. Multivariate logistic regression analysis was performed to find factors associated with HCV infection or unaware HCV infection. P values less than 0.05 were considered to be significant. Finally, data handling and analysis were performed with SPSS software for Windows, version 17.0 (SPSS Inc., Chicago, IL).

## Results

### 1. Baseline characteristics

Of the 40000 estimated subjects in the screening couties, 1439 residents (3.6%) participated in the intervention voluntarily in the screening period. After exclusion of those residents with positive HBsAg (n = 131) and dual positivity of HBsAg and anti-HCV Ab (n = 23), non-HCV infection (both negative for HBsAg and anti-HCV and mono-HCV infection (positive anti-HCV) were observed in 1077 and 194 residents, respectively. Non-HCV infected residents exhibited the features of younger age, lower BMI, less WC and HC, higher ALT and AST levels, less hypertension and DM, higher values of controlled attenuation parameter (CAP), and lower values of liver stiffness than HCV infected residents “Table 1”. By multivariate logistic regression analysis, HCV infected residents had significant older age (p<0.001), higher ALT levels (p<0.001), higher liver stiffness (p = 0.010), and lower CAP values (p<0.001).

**Table 1:** Comparisons of demography of residents with status of HCV infection.

	Univariate			Multivariate		
	Non-HCV (n = 1077)	HCV (n = 194)	p values	OR	95% CI	p values
Age (yrs)	54.8 ± 16.2	65.3 ± 11.8	<0.001	1.052	1.037~1.068	<0.001
≤ 40	236	5	<0.001			
40 ~ 50	152	13				
50 ~ 60	252	41				
60 ~ 70	236	69				
>70	20	66				
Gender (M/F)	440/637	77/117	0.241			
BMI (kg/m^2^)	27.0 ± 13.0	24.6 ± 5.0	0.008	1.000	0.996~1.004	0.998
Waist circumference (cm)	82.0 ± 12.1	86.1 ± 10.1	<0.001	1.019	0.996~1.042	0.112
Hip circumference (cm)	95.7 ± 9.7	97.2 ± 8.5	0.038	1.009	0.996~1.037	0.528
GOT (U/L)	27.0 ± 13.0	44.0 ± 57.3	<0.001	0.011	0.995~1.028	0.163
GPT (U/L)	24.0 ± 17.3	45.0 ± 85.8	0.002	1.025	1.012~1.038	<0.001
Liver stiffness (kPa)	5.1 ± 5.2	7.7 ± 6.8	<0.001	1.037	1.009~1.067	0.010
CAP (dB/m)	240.1 ± 54.4	227.8 ± 49.0	<0.001	0.988	0.984~0.992	<0.001
NAFLD (yes/no)	545/532	89/105	0.313			
Smoking (yes/no)	99/967	26/168	0.271			
Moderate alcohol consumption (yes/no)	68/998	16/178	0.435			
Hypertension (yes/no)	257/809	75/119	<0.001	1.287	0.856~1.936	0.225
Diabetes mellitus (yes/no)	118/947	32/162	0.007	0.834	0.494~1.406	0.495
Dyslipidemia (yes/no)	119/945	23/171	0.918			
Hyperuricemia (yes/no)	40/1026	11/183	0.390			
Stroke history (yes/no)	7/1059	5/189	0.057			
Heart diseases (yes/no)	77/988	20/174	0.38			
Kidney diseases (yes/no)	14/1052	5/189	0.172			
Thyroid diseases (yes/no)	35/1031	5/189	1.000			
Family History of liver disease (yes/no)	219/845	48/146	0.392			

Missing data in non-HCV subjects was observed.

The overall prevalence of HCV infection was 13.5%. They were tended to be in older age with 135 (69.6%) more than 60 years and female predominant. Table 2 shows the baseline characteristics of the 194 HCV infected participants. Diabetes mellitus, dyslipidemia, and hypertension were observed in 16.5%, 11.9%, and 38.7% of residents, respectively.

**Table 2:** Characteristics of positive anti-HCV Ab subjects with known or unaware HCV infection.

		Univariate				Multivariate		
	All (n = 194)	Known (n = 126)	Unaware (n = 68)	p values	Analyzed Patients	OR	95% CI	p values
Age (yrs)	65.3 ± 11.8	63.1 ± 10.8	69.2 ± 12.6	0.001	194	1.028	0.996~1.0604	0.089
≤ 40	5	3	2	<0.001				
40 ~ 50	13	11	2					
50 ~ 60	41	31	10					
60 ~ 70	69	52	17					
>70	66	29	37					
Gender (M/F)	77/177	51/75	26/42	0.761				
BMI (kg/m^2^)	24.6 ± 5.0	25.6 ± 4.1	24.0 ± 3.8	0.008	194	0.933	0.851~1.023	0.142
Waist circumference (cm)	86.1 ± 10.1	87.2 ± 10.7	84.0 ± 8.6	0.024	194	0.987	0.938~1.037	0.597
Abnormal WC (yes/no)	101/93	71/55	30/38	0.136				
Hip circumference (cm)	97.2 ± 8.5	98.4 ± 8.4	95.2 ± 8.6	0.014	194	1.014	0.964~1.066	0.594
WHR	0.89 ± 0.08	0.89 ± 0.08	0.89 ± 0.08	0.886				
Abnormal WHR	76/118	47/80	29/38	0.400				
GOT (U/L)	44.0 ± 57.3	43.5 ± 56.0	45.3 ± 60.7	0.831				
GPT (U/L)	45.0 ± 85.8	40.7 ± 34.4	53.6 ± 138.1	0.452				
Liver stiffness (kPa)	7.7 ± 6.8	8.6 ± 8.3	6.1 ± 2.4	0.002	191	0.876	0.782~0.981	0.022
CAP (dB/m)	227.8 ± 49.0	229.4 ± 48.4	214.3 ± 48.4	0.043	190	0.998	0.990~1.005	0.531
NAFLD (yes/no)	89/105	66/60	23/45	0.013	194	0.789	0.364~1.710	0.548
Smoking (yes/no)	26/168	19/107	7/61	0.351				
Moderate alcohol consumption (yes/no)	16/178	11/115	5/63	0.739				
Hypertension (yes/no)	75/119	49/77	26/42	0.929				
Diabetes mellitus (yes/no)	32/162	20/106	12/56	0.751				
Dyslipidemia (yes/no)	23/171	15/111	8/60	0.977				
Hyperuricemia (yes/no)	11/183	9/117	2/66	0.334				
Stroke history (yes/no)	5/189	4/122	1/67	0.659				
Heart diseases (yes/no)	20/174	6/120	15/54	0.001	194	6.082	1.963~18.839	0.002
Kidney diseases (yes/no)	5/189	3/123	2/66	1.000				
Thyroid diseases (yes/no)	5/189	3/123	2/66	1.000				
Family History of liver disease (yes/no)	48/146	40/86	8/68	0.002	194	0.291	0.113~0.750	0.011

### 2. Comparisons between residents with already known or previously unaware HCV infection

Among the 194 residents with HCV infection, 68 (35.1%) did not have the awareness of HCV infection before and 119 (61.3%) exhibited HCV viremia. We analyzed the difference of characteristics between already known HCV infected subjects and previously unaware HCV infected subjects. Residents with previously unaware HCV infection exhibited significant older age (p = 0.001), lower BMI (p = 0.008), less waist and hip circumferences (p = 0.024 and p = 0.014), lower values of liver stiffness (p = 0.002) and controlled attenuation parameter (CAP; p = 0.043), less prevalence of NAFLD (p = 0.013) and heart diseases (p = 0.002), and less cluster of family history of liver diseases (p = 0.002) “Table 2”. Comparison was also performed in the 119 viremic residents, including 76 (62.2%) already known HCV infection and 45 (37.8%) previously unaware HCV infection. Similar factors were observed and showed in Table 3.

**Table 3:** The characteristics of HCV viremic patients with already known or unaware HCV infection.

		Univariate				Multivariate		
	All (n = 119)	Known (n = 74)	Unaware (n = 45)	p values	Analyzed patients	OR	95% CI	p values
Age (years)	66.3 ± 12.4	64.1 ± 14.3	69.7 ± 13.5	0.025	119	0.987	0.952~1.024	0.485
≤ 40	2	1	1	0.134				
40 ~ 50	9	7	2					
50 ~ 60	22	14	8					
60 ~ 70	39	29	10					
>70	47	23	24					
Gender (M/F)	72/47	28/46	19/26	0.635				
BMI (kg/m^2^)	24.7 ± 4.7	25.5 ± 4.3	24.0 ± 3.7	0.045	119	1.059	0.951~1.180	0.296
Waist circumference (cm)	85.7 ± 10.5	86.4 ± 11.4	84.4 ± 9.0	0.292				
Abnormal WC	56/63	32/42	24/21	0.261				
Hip circumference (cm)	97.1 ± 9.0	98.0 ± 9.2	95.8 ± 8.6	0.203				
WHR	0.88 ± 0.08	0.88 ± 0.09	0.88 ± 0.07	0.994				
Abnormal WHR	48/71	30/44	18/27	0.984				
GOT (U/L)	50.0 ±67.3	51.5 ± 70.3	47.9 ± 63.6	0.778				
GPT (U/L)	55.1 ± 104.6	50.7 ± 40.9	63.1 ± 163.4	0.533				
Liver stiffness (kPa)	8.2 ± 7.5	9.3 ± 9.2	6.2 ± 2.0	0.006	117	1.187	1.029~1.369	0.019
CAP (dB/m)	219.9 ± 49.8	226.7 ± 50.1	207.5 ± 47.6	0.045	117	1.004	0.995~1.014	0.352
NAFLD (yes/no)	51/68	37/37	14/31	0.043				
Smoking (yes/no)	18/101	13/61	5/40	0.340				
Moderate alcohol consumption (yes/no)	11/108	7/67	4/41	0.917				
Hypertension (yes/no)	43/78	28/46	15/30	0.620				
Diabetes Mellitus (yes/no)	22/97	14/60	8/37	0.876				
Dyslipidemia (yes/no)	8/111	5/69	3/42	1.000				
Hyperuricemia (yes/no)	6/113	6/68	0/45	0.082				
Stroke history (yes/no)	4/115	3/71	1/44	1.000				
Heart diseases (yes/no)	12/107	3/71	9/36	0.009	119	0.112	0.021~0.603	0.011
Kidney diseases (yes/no)	3/116	1/73	2/49	0.556				
Thyroid diseases (yes/no)	3/116	2/72	1/44	1.000				
Family History of liver disease (yes/no)	31/88	24/50	7/38	0.042	119	2.390	0.787~7.263	0.124

We further performed multivariate logistic regression analysis to find factors associated with the unaware HCV infection. Less fibrosis severity (OR 0.876, 95% CI 0.782~0.981, p = 0.022), more prevalent of heart diseases (OR 6.082, 95% CI 1.963~18.839, p = 0.002), and less cluster of family history of liver diseases (OR 0.291, 95% CI 0.113~0.750, p = 0.011) were the independent factors “Table 2”. For HCV viremic residents, unaware HCV infected residents were associated with less fibrosis severity (OR 1.187, 95% CI 1.029~1.369, p = 0.019) and more prevalent of heart diseases (OR 0.112, 95% CI 0.021~0.603, p = 0.011) “Table 3”.

### 3. Insights of liver diseases in HCV infected residents

**Fig 1:**
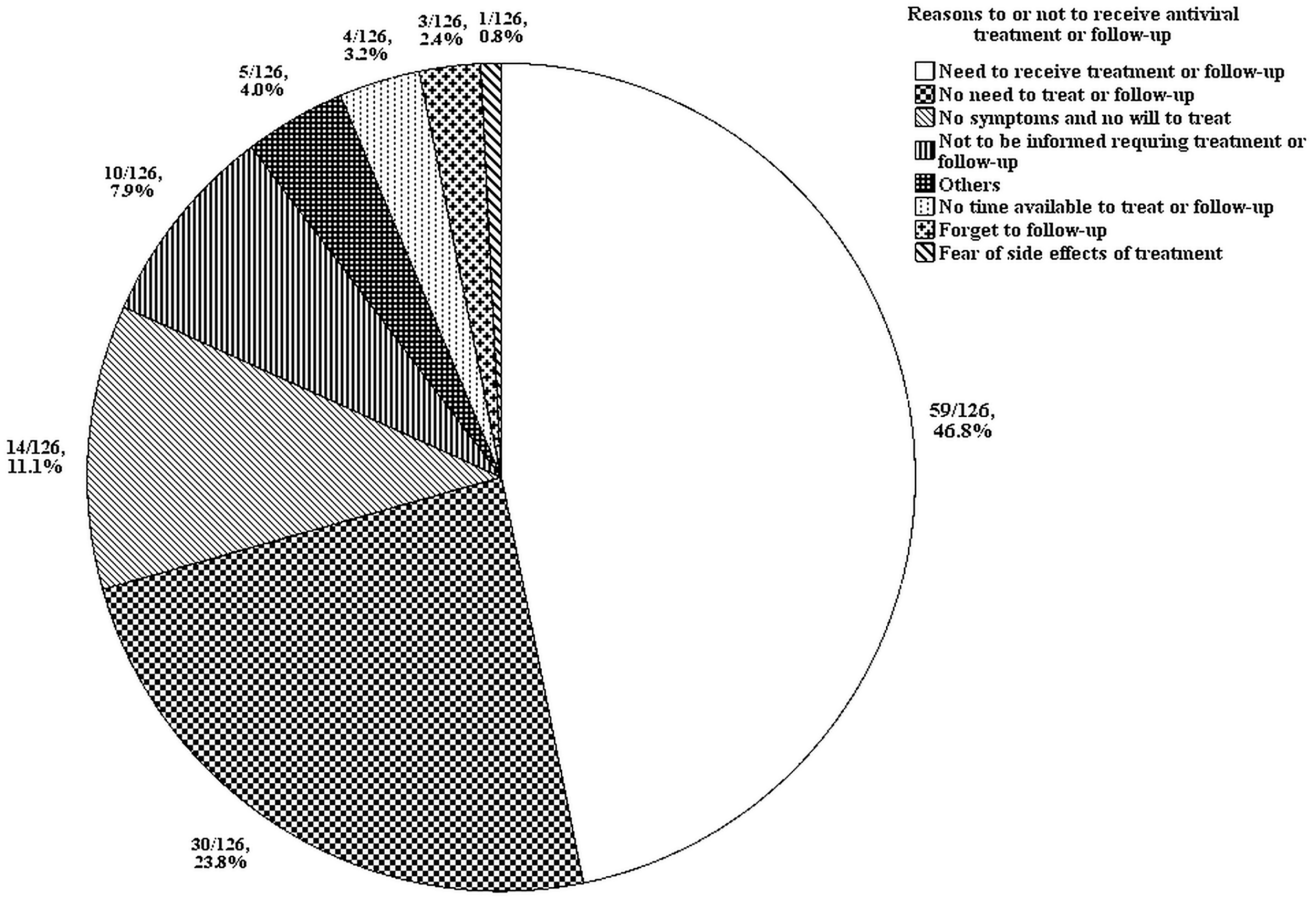
The distribution of disease insights in 126 already known HCV infected residents.

For understanding the altitude or knowledge of facing HCV infection, we evaluated the insights of liver diseases in 126 already known HCV infected residents. Only 59 (46.8%) of them had current or history of antiviral treatment or regular follow-up at hospital. Six patients received pegylated interferon/ribavirin treatment and achieved sustained virological response. In remaining residents, 30 (23.8%) did not have any concept of treatment or follow-up; 14 (11.1%) did not have any willing to treatment or follow-up due to no symptoms; 10 (7.9%) was informed not requiring to receive treatment or follow-up by general physicians. Fig 1 shows the detail distribution of disease insights.

## Discussion

This screening intervention is a community-based approach to discover or screen residents in HCV and HBV endemic villages in southern Taiwan. In this study, higher prevalence of HCV infection (13.5%) than that of general population in Taiwan indicated that these villages were HCV endemic area [14,15]. Not surprisingly, HCV infected residents were older and more significant liver injury than non-HCV infected residents. Of the HCV infected residents, about one third of them were unaware of infection before participating in screening intervention. They exhibited different features from those already known HCV infected residents in older age, less BMI, milder liver fibrosis and less prevalence of NAFLD, and less family cluster of liver diseases. The target population of this study is quite different from previous reports [16,17]. Residents lived in known HCV endemic and remote villages of Taiwan would be a distinct and special population. Relatively poor healthy knowledge and resources, long transportation distance to hospital, predominantly older people, and lower socioeconomic status are the main characteristics of residents in remote villages of southern Taiwan. In addition, there was a lack of report that described the comparison between already known and unaware HCV infected residents in community.

The unaware HCV infected subjects is a distinct and important population nowadays. We found that 35% of residents were unaware of HCV infection before participating in screening intervention which was higher than previous reports from Taiwan (about 15%) [18,19]. The difference mainly based on the screening sites as our screening intervention focused on remote villages whereas other screening intervention focused on urban and rural regions [18,19]. The proportion and population of unaware of HCV infection is also different from a report from Western country [20]. This result indicated that a substantial group of HCV infected residents remained undiscovered even after rigorous screening activities conducted by local government or non-governmental organizations (NGOs) for many years in remote villages of Taiwan. There are many reasons that the previously unaware infected residents could be discovered in this study. In our screening intervention, we contacted local representatives of villages to join and to play an active role in encouraging residents to participate in activities, including broadcasting screening activity news and spreading leaflets in villages. In addition, the remote villages often exhibit relatively poor health resources and long distance to reach hospital. Screening activities held locally that may enhance the incentives of residents to participate in. This screening model is different from those reported before and may be considered as a valuable option for HCV endemic regions.

Understanding the characteristics of previously unaware infected residents or subjects is an important issue that seldom addressed before. In this study, residents with previously unaware HCV infection exhibited features of mild severity of liver diseases, higher rates of heart diseases, and less family cluster of liver diseases. The mild disease severity by measuring the liver stiffness implicated that this kind of infected subjects probably were less symptomatic and then consequently resulted in less opportunities to seek for medical aids. Disease severity may also associate with genetic background. Lines of evidence showed that *IL28B* and *PNPLA3* polymorphism were associated with advanced liver diseases and steatosis in chronic hepatitis C [21–23]. Although genetic factors such as PNPAL3 or others did not survey in this study, genetic predisposing cannot be totally excluded.

Residents with family history of liver diseases theoretically gain more health knowledge and have more incentive to test whether they got hepatitis virus infection. Infected family members may also urge other members to evaluate the hepatitis virus infection. Hence, family with current or history of liver diseases may cause a positive feedback circus within family. Unaware of HCV infected residents seem to be lack of these external effects that probably make them persistently undiscovered.

Unaware HCV infection residents presented with more prevalence of heart diseases. The only observed risk factor in this study was older age. There was no difference in the prevalence of diabetes mellitus, hypertension, smoking history, and alcohol consumption. However, already known HCV infected residents had risk factors such as higher waist/hip circumferences and more NAFLD that both could associate with the occurrence of heart diseases [24,25]. At present, we could not explain this association well.

In this study, less than half of infected residents have been treated or followed at hospital in community of southern Taiwan. One third of residents had insights of no concept or no willing to treat or follow-up. All these three factors indicated that both heath knowledge and linkage to medical care of HCV infected residents were poor in remote villages. Barriers that may associate with these features include long transportation distance to referral or medical centers, poor health resources of villages to evaluate disease severity, lack of call-back or follow-up system, and insufficient manpower including healthy workers and public officers to educate or actively refer these infected residents. The best strategies to HCV elimination should put more efforts to minimize the gap between screening and active treatment [26], especially in high effectiveness DAA era. The actions shall include broadening screening coverage in known HCV endemic villages, cooperating with local government or NGOs to maximize screening resources and to actively refer for medical care, and educating general physicians and residents of update knowledge of liver diseases and treatments.

There are limitations of this study. Although this is a prospective study, we did not store enough blood samples to perform genetic or additional biochemical tests. The findings of this study were based on the population of HCV endemic remote villages in Taiwan and may not be applied to general population.

## Conclusions

In conclusion, unaware of HCV infected residents represented a substantial and distinct HCV infected population with obscure characteristics in community. To augment the linkage of medical care to known HCV infected residents and discover unaware infected residents, further work including how to enhance screening coverage, methods to identify liver disease severity, efforts to propagate adequate or update knowledge of liver diseases, and establish efficient call-back or follow-up system should be addressed.

## Supporting information

S1 File The questionnaire in Chinese. (DOC)

S2 File The questionnaire in English. (DOC)

S3 File The analyzed date set in SPSS format. (SAV)

## Abbreviations

ALT: alanine aminotransferase
Anti-HCV: anti-hepatitis C virus antibody
AST: aspartate aminotransferase
CAP: controlled attenuation parameter
CHC: chronic hepatitis C
DAA: direct acting antiviral agents
HBsAg: hepatitis B surface antigen
HCV: hepatitis C virus
kPa: kilopascals
NAFLD: non-alcoholic fatty liver disease
NGO: non-governmental organization

## References

[1] WHO. Guidelines for the screening, care and treatment of persons with hepatitis Cinfection. 2014. http://www.who.int/hiv/pub/hepatitis/hepatitis-c-guidelines/en/ (accessed Sept 3, 2014). 25535634

[2] Gower E, Estes C, Blach S, Razavi-Shearer K, Razavi H. Global epidemiology and genotype distribution of the hepatitis C virus infection. J Hepatol. 2014;61 Suppl:S45–57. 25086286 10.1016/j.jhep.2014.07.027

[3] Thein HH, Yi QL, Dore GJ, Krahn MD. Estimation of stage-specific fibrosis progression rates in chronic hepatitis C virus infection: a meta-analysis and meta-regression. Hepatology 2008;48:418–431. doi: 10.1002/hep.22375 18563841 10.1002/hep.22375

[4] Westbrook RH, Dusheiko G. Natural history of hepatitis C. J Hepatol. 2014;61 Suppl;S58–68. 25443346 10.1016/j.jhep.2014.07.012

[5] Poynard T, Bedossa P, Opolon P. Natural history of liver fibrosis progression in patients with chronic hepatitis C. Lancet 1997;349:825–832. 9121257 10.1016/s0140-6736(96)07642-8

[6] Negro F, Forton D, Craxi A, Sulkowski MS, Feld JJ, Manns MP. Extrahepatic morbidity and mortality of chronic hepatitis C. Gastroenterology 2015;149:1345–1360. doi: 10.1053/j.gastro.2015.08.035 26319013 10.1053/j.gastro.2015.08.035

[7] Morgan TR, Ghany MG, Kim H-Y, Snow KK, Shiffman ML, De Santo JL, et al, HALT-C Trial Group. Outcome of sustained virological responders with histologically advanced chronic hepatitis C. Hepatology 2010;52:833–844. doi: 10.1002/hep.23744 20564351 10.1002/hep.23744 PMC2932862

[8] George SL, Bacon BR, Brunt EM, Mihindukulasuriya KL, Hoffmann J, Di Bisceglie AM. Clinical, virologic, histologic, and biochemical outcomes after successful HCV therapy: a 5-year follow-up of 150 patients. Hepatology 2009;49:729–738. doi: 10.1002/hep.22694 19072828 10.1002/hep.22694 PMC2731713

[9] Curry MP, Tapper EB, Bacon B, Dieterich D, Flamm SL, Guest L, et al Effectiveness of 8- or 12-weeks of ledipasvir and sofosbuvir in real-world treatment-naïve, genotype 1 hepatitis C infected patients. Aliment Pharmacol Ther 2017;46:540–548. doi: 10.1111/apt.14204 28691377 10.1111/apt.14204

[10] Gane EJ, Pianko S, Roberts SK, Thompson AJ, Zeuzem S, Zuckerman E, et al Safety and efficacy of an 8-week regimen of grazoprevir plus ruzasvir plus uprifosbuvir compared with grazoprevir plus elbasvir plus uprifosbuvir in participants without cirrhosis infected with hepatitis C virus genotypes 1, 2, or 3 (C-CREST-1 and C-CREST-2, part A): two randomised, phase 2, open-label trials. Lancet Gastroenterol Hepatol. 2017 doi: 10.1016/S2468-1253(17)30159-0 10.1016/S2468-1253(17)30159-0 28802816

[11] Global Hepatitis Report 2017. Geneva: World Health Organization; 2017.

[12] Cheng PN, Chiu YC, Chiu HC, Chien SC. The Application of Liver Stiffness Measurement in Residents Without Overt Liver Diseases Through a Community-Based Screening program. Medicine 2016;95:e3193 doi: 10.1097/MD.0000000000003193 27015215 10.1097/MD.0000000000003193 PMC4998410

[13] Hamaguchi M, Kojima T, Itoh Y, Harano Y, Fujii K, Nakajima T, et al The severity of ultrasonographic findings in nonalcoholic fatty liver disease reflects the metabolic syndrome and visceral fat accumulation. Am J Gastroenterol 2007;102:2708–2715. doi: 10.1111/j.1572-0241.2007.01526.x 17894848 10.1111/j.1572-0241.2007.01526.x

[14] Chen CH, Yang PM, Huang GT, et al Estimation of seroprevalence of hepatitis B virus and hepatitis C virus in Taiwan from a large-scale survey of free hepatitis screening participants. J Formos Med Assoc 2007; 106:148–155. doi: 10.1016/S0929-6646(09)60231-X 17339159 10.1016/S0929-6646(09)60231-X

[15] Yang JF, Lin CI, Huang JF, et al Viral hepatitis infections in southern Taiwan: a multicenter community-based study. Kaohsiung J Med Sci 2010; 26:461–469. doi: 10.1016/S1607-551X(10)70073-5 20837342 10.1016/S1607-551X(10)70073-5 PMC11916219

[16] Konerman MA, Thomson M, Gray K, Moore M, Choxi H, Seif E, et al Impact of an Electronic Health Record Alert in Primary Care on Increasing Hepatitis C Screening and Curative Treatment for Baby Boomers. Hepatology. 2017;17 doi: 10.1002/hep.29362 28714196 10.1002/hep.29362 PMC5696058

[17] Castrejón M, Chew KW, Javanbakht M, Humphries R, Saab S, Klausner JD. Implementation of a Large System-Wide Hepatitis C Virus Screening and Linkage to Care program for Baby Boomers. Open Forum Infect Dis 2017;4:ofx109 doi: 10.1093/ofid/ofx109 28752101 10.1093/ofid/ofx109 PMC5527269

[18] Liver Disease Prevention and Treatment Research Foundation. National hepatitis screening report. Liver Disease prevention and Treatment Journal. 2005;28:11–13. (in Chinese)

[19] Kuo YH, Chen PF, Wang JH, Chang KC, Kee KM, Tsai MC, et al Comparison Stratagems of Post-Screening Management of Anti-HCV-Positive Community Residents: Simple Notification, Active Referral, or Accessible Medical Care. PLoS One. 2015;10:e0126031 doi: 10.1371/journal.pone.0126031 eCollection 2015 25970487 10.1371/journal.pone.0126031 PMC4430481

[20] Prevost TC, Presanis AM, Taylor A, Goldberg DJ, Hutchinson SJ, De Angelis D. Estimating the number of people with hepatitis C virus who have ever injected drugs and have yet to be diagnosed: an evidence synthesis approach for Scotland. Addiction 2015;110:1287–300. doi: 10.1111/add.12948 25876667 10.1111/add.12948 PMC4744705

[21] Fan JH, Xiang MQ, Li QL, Shi HT, Guo JJ. PNPLA3 rs738409 Polymorphism Associated with Hepatic Steatosis and Advanced Fibrosis in Patients with Chronic Hepatitis C Virus: A Meta-Analysis. Gut Liver. 2016;10:456–63. doi: 10.5009/gnl15261 26419236 10.5009/gnl15261 PMC4849700

[22] Trépo E, Pradat P, Potthoff A, Momozawa Y, Quertinmont E, Gustot T, et al Impact of patatin-like phospholipase-3 (rs738409 C&gt;G) polymorphism on fibrosis progression and steatosis in chronic hepatitis C. Hepatology. 2011;54:60–69. doi: 10.1002/hep.24350 21488075 10.1002/hep.24350

[23] Tamaki N, Kurosaki M, Higuchi M, Takada H, Nakakuki N, Yasui Y, et al Genetic Polymorphisms of IL28B and PNPLA3 Are Predictive for HCV Related Rapid Fibrosis Progression and Identify Patients Who Require Urgent Antiviral Treatment with New Regimens. PLoS One. 2015;10:e0137351 doi: 10.1371/journal.pone.0137351 26352693 10.1371/journal.pone.0137351 PMC4564246

[24] Dalton M, Cameron AJ, Zimmet PZ, Shaw JE, Jolley D, Dunstan DW, Welborn TA on behalf of the AUSDIAB steering committee. Waist circumference, waist—hip ratio and body mass index and their correlation with cardiovascular disease risk factors in Australian adults. J Intern Med 2003;254:555–563. 14641796 10.1111/j.1365-2796.2003.01229.x

[25] Francque SM, van der Graaff D, Kwanten WJ. Non-alcoholic fatty liver disease and cardiovascular risk: Pathophysiological mechanisms and implications. J Hepatol 2016;65:425–443. doi: 10.1016/j.jhep.2016.04.005 27091791 10.1016/j.jhep.2016.04.005

[26] Hagan LM, Schinazi RF. Best strategies for global HCV eradication. Liver Int. 2013;33 Suppl 1:68–79. 23286849 10.1111/liv.12063 PMC4110680

