# Supplementary material for: The characteristics of residents with unawareness of hepatitis C virus infection in community
Source: PLoS One. 2018 Feb 22;13(2):e0193251. doi: 10.1371/journal.pone.0193251 (PMC5823433; doi:10.1371/journal.pone.0193251)
Supplement: S1 File — (DOC) [file pone.0193251.s001.doc]

|  |
| --- |

編號:

日期:

**一、**基本資料

| 姓名: 生日: 身分證字號:  電話: 地址:  教育程度: □1.不識字 □2.國小 □3.初中 □4.高中(職) □5.大學(專) □6.碩士 □7.博士  目前職業： □1.學生 □2.就業中 □3.待業中 □4.退休 □5.家庭主婦  身高: cm 體重: kg 腰圍: cm 臀圍: cm 血壓： |
| --- |

**二、個人健康資料**

| 1、抽菸情形 £1.無 £2.有 (持續 年、 支/天) £3.偶爾 £4.曾經，已戒 年  2、喝酒情形 £1.無 £2.有 (持續 年、 次/週) £3.偶爾 £4.曾經，已戒 年  3、您是否有下述疾病?  □0.沒有 £1.高血壓 £2.糖尿病 £3.血脂肪過高 £4.尿酸過高 £5.腦中風  £6.心臟病 £7.腎臟病 £8.甲狀腺疾病 £9.肝病 £10. 不詳  £11. 其他 |
| --- |

三、家族史

| 1、家族成員是否有肝臟疾病 ?  □1.無  □2.有 ( □1.B型肝炎 □2.C型肝炎 □3.肝硬化 □4.肝癌 □5.其他 ) |
| --- |

**四 -B型、C型肝炎檢查和疫苗**

**B型肝炎檢查和疫苗的問題**

1. 您以前是否做過B型肝炎檢查？

（請注意B型肝炎檢查並非肝功能檢查）

□ 1. 有 (續答第2、3題)

□ 2. 沒有 (續答第5題)

1. 您何時去做B型肝炎檢查的？

□ 1. 今年

□ 2. 一至五年前

□ 3. 五至十年前

□ 4. 十年或更多年以前

1. 檢查結果是什麼？

□ 1. 感染了B型肝炎(續答第4題)

□ 2. 沒有感染B型肝炎(續答第5題)

□ 3. 不知道

1. 如果您有感染B肝，請問您有無接受治療或者後續檢查的原因是什麼？**[可複選]**

□ 1. 我有接受治療或者後續檢查，

是__________科的醫生

□ 2. 醫師無告知需要接受治療或者後續檢查

□ 3. 擔心B肝藥物治療的副作用

□ 4. 太忙

□ 5. 以為健保沒給付需要自費

□ 6. 無症狀無意願接受治療

□ 7. 忘了.

□ 8. 其它：請註明 ________________

1. 您是否接種過B型肝炎疫苗？

□ 1. 有

□ 2. 沒有

**C型肝炎檢查和治療問題**

1. 您以前是否做過Ｃ型肝炎檢查？

（請注意Ｃ型肝炎檢查並非肝功能檢查）

□ 1. 有 (續答第7、8題)

□ 2. 沒有(跳至第五部分做答)

1. 您何時去做Ｃ型肝炎檢查的？

□ 1. 今年

□ 2. 一至五年前

□ 3. 五至十年前

□ 4. 十年或更多年以前

1. 檢查結果是什麼？

□ 1. 感染了Ｃ型肝炎(續答第9題)

□ 2. 沒有感染Ｃ型肝炎(跳至第五部分做答)

□ 3. 不知道(跳至第五部分做答)

1. 如果您有感染C肝，請問您沒有接受治療或者後續檢查的原因是什麼？ **[可複選]**

□ 1. 我有接受治療或者後續檢查，

是__________科的醫生

□ 2. 醫師無告知需要接受治療或者後續檢查

□ 3. 擔心C肝藥物治療的副作用

□ 4. 太忙

□ 5. 以為健保沒給付需要自費

□ 6. 無症狀無意願接受治療

□ 7. 忘了.

□ 8. 其它：請註明 ____________

**五–病人，醫生，資源**

1、請問您會去哪裡看病？**[可複選]** (可複選)

□ 1.衛生所 □ 2.診所 □ 3.醫院 □ 4.急診室 □ 5.中醫 □ 6.一般藥局

□ 7. 其他，請說明____________

2、有沒有一個您固定去看的醫生？

□ 1. 有 □ 2. 沒有

3、最近三個月，您是否有使用電台廣告購買的藥物？

□ 1. 有，包括: ________________________ □ 2. 沒有

**六、 B、C型肝炎知識—**

**(一) B型肝炎傳染途徑**

**1.您認為下列是否為B肝的傳染途徑？ （請在每一行勾選一個答案）**

|  | 是 | 不是 | 不知道 |
| --- | --- | --- | --- |
| 1. 出生的時候從母親傳染給嬰兒 |  |  |  |
| 1. 被患B肝的母親哺乳 |  |  |  |
| 1. 和B肝患者一起用餐 |  |  |  |
| 1. 吃了B肝患者準備的食物 |  |  |  |
| 1. 吃B肝患者咀嚼過的食物 |  |  |  |
| 1. 和B肝患者共用牙刷 |  |  |  |
| 1. 和B肝患者共用剃刀 |  |  |  |
| 1. 被B肝患者咳嗽或者打噴嚏噴到 |  |  |  |
| 1. 和B肝患者牽手 |  |  |  |
| 1. 和B肝患者發生性行為 |  |  |  |

**2. B肝病毒感染的後果: 以下的敘述您認為…**

|  | 是 | 不是 | 不知道 |
| --- | --- | --- | --- |
| 1. B肝患者會一輩子都有B肝 |  |  |  |
| 1. 沒有妥善追蹤治療B型肝炎會導致肝癌和肝硬化 |  |  |  |
| 1. B肝不易治癒，但是病情可以得到控制 |  |  |  |
| 1. 大部分的慢性B肝患者不會有症狀 |  |  |  |
| 1. 看起來健康的B肝患者仍然可以傳播B肝 |  |  |  |

**(二) Ｃ型肝炎傳染途徑**

**1、您認為下列是否為Ｃ肝的傳染途徑？**（請在每一行勾選一個答案）

|  | 是 | 不是 | 不知道 |
| --- | --- | --- | --- |
| 1. C肝可以在出生的時候從已感染的母親傳染給嬰兒 |  |  |  |
| 2.被C肝患者捐血可以導致C肝感染 |  |  |  |
| 3.跟有C肝的人一起工作可以導致C肝感染 |  |  |  |
| 4.被有著C肝患者的血的針頭或尖銳物品紮到可以導致C肝感染 |  |  |  |
| 5.就算次數不多，注射非法藥物可以導致C肝感染 |  |  |  |
| 6.跟C肝患者握手可以導致C肝感染 |  |  |  |
| 7.跟C肝患者親吻可以導致C肝感染 |  |  |  |
| 8.跟C肝患者發生性行為可以導致C肝感染 |  |  |  |

**2. C肝病毒感染的後果: 以下的敘述您認為…**

|  | 是 | 不是 | 不知道 |
| --- | --- | --- | --- |
| 1.如果感染了Ｃ肝病毒，很有可能會一輩子都感染 |  |  |  |
| 2.沒有妥善追蹤治療C型肝炎會導致肝癌和肝硬化 |  |  |  |
| 3.C肝可以被治癒 |  |  |  |
| 4.大部分的C肝患者不會有症狀 |  |  |  |
| 5.看起來健康的C肝患者仍然可以傳播C肝 |  |  |  |

**七 – 其他影響因素**

請問您是否曾經有過下列經驗？（請在每一行勾選一個答案）

|  | 有 | 沒有 | 不知道 |
| --- | --- | --- | --- |
| 1.刺青 |  |  |  |
| 2.輸血 |  |  |  |
| 3.施打毒品 |  |  |  |
| 4.手術 |  |  |  |
| 5.共用牙刷 |  |  |  |
| 6.共用剃刀 |  |  |  |
| 7.共用針頭 |  |  |  |
| 8.針灸、拔罐 |  |  |  |
| 9.治療牙齒 |  |  |  |

***個人資料保護管理:***

*在符合個人資料保護法第8條規定下，本次篩檢活*

*動為提供您更完整的醫療評估、診斷與建議，將蒐*

*集您的個人辨識資料、個人與身體描述、習慣、家庭情形、職業及健康紀錄等個資。您可自由選擇是否提供，如不提供將無法針對您具體情況做出正確、有利的診治。您有權依同法第3條規定要求查閱、補充或更正資料，並得隨時請求停止蒐集或利用之。*

*簽名/日期:*
