# Supplementary material for: The characteristics of residents with unawareness of hepatitis C virus infection in community
Source: PLoS One. 2018 Feb 22;13(2):e0193251. doi: 10.1371/journal.pone.0193251 (PMC5823433; doi:10.1371/journal.pone.0193251)
Supplement: S2 File — (DOC) [file pone.0193251.s002.doc]

|  |
| --- |

Number:

Date:

**Ⅰ、Basic information**

| Name: Date of birth: ID card number:  Telephone: Address:  Education level: □1.Not literate □2. Junior high school. □ 3. Junior high school □ 4. high school (post)  □5. University (special) □ 6. Master □ 7. doctor  Current Occupation: □1. Student □2. Employment □3. Unemployed □4. Retired □5. Housewife  Height: cm Weight: kg Waist: cm Hips: cm Blood pressure: |
| --- |

**Ⅱ、Personal Health Information**

| 1, Smoking ：□ 1.No □ 2.Yes ( for years, Branch / day ) □ 3.Occasionally  □ 4. Once, have quit years  2, Drinking ：□ 1. No □ 2. Yes ( continuous years, times / week) □ 3. Occasionally  □ 4. Once, have quit years  3、Do you have the following diseases?  □ 0. No □ 1. Hypertension □ 2. Diabetes □ 3. Hyperlipemia □ 4. Hyperuria □ 5. Stroke     □ 6. Heart disease □ 7. Kidney disease □ 8. Thyroid disease □ 9. Liver disease  □ 10. Unknown □ 11.Other |
| --- |

**Ⅲ、Family history**

| 1、Does your family have liver disease?  □ 1. None  □ 2. Yes ( □ 1.HBV □ 2.HCV □ 3. Cirrhosis □ 4. Liver cancer □ 5. Others ) |
| --- |

**Ⅳ、Hepatitis B, hepatitis C examination and vaccine**

**Hepatitis B examination and vaccine problems**

1. Have you done a Hepatitis B test before?

(Please note that hepatitis B is not a liver function test)

   □ 1. Yes (to answer questions 2 and 3)

   □ 2. No (continued Q5)

1. When do you check hepatitis B test?

   □ 1. This year

   □ 2. One to five years ago

   □ 3. Five to ten years ago

   □ 4. Ten years or more ago

1. What is the test result?

   □ 1. Infection with hepatitis B (continued Q4)

   □ 2. No infection with hepatitis B (continued Q5)

   □ 3. I do not know

1. If you have B liver infection, what are the

reasons for your treatment or follow-up? [Multiple choice]

□ 1. I have received treatment or follow-up examination ,Is a __________ section of the doctor

□ 2. The physician needs no treatment or follow-up

examination without notification

□ 3. Worried about the side effects of B liver drug

treatment

□ 4. Too busy

□ 5. I did not pay for health insurance at their own

expense

□ 6. Asymptomatic unwilling to receive treatment

□ 7. Forgot.

□ 8. Others: Please specify

1. Did you get hepatitis B vaccine?
   - 1. Yes
   - 2.No

**Hepatitis C examination and vaccine problems**

1. Have you done a Hepatitis C test before?

(Please note that hepatitis C is not a liver function test)

□ 1. Yes (to answer questions 7 and 8 )

   □ 2. No (continued Q5)

1. When do you check hepatitis C test?

   □ 1. This year

   □ 2. One to five years ago

   □ 3. Five to ten years ago

   □ 4. Ten years or more ago

1. What is the test result?

   □ 1. Infection with hepatitis C (continued Q4)

   □ 2. No infection with hepatitis C (continued Q5)

   □ 3. I do not know

1. If you have C liver infection, what are the

reasons for your treatment or follow-up? [Multiple choice]

□ 1. I have received treatment or follow-up examination , Is a __________ section of the doctor

□ 2. The physician needs no treatment or follow-up

examination without notification

□ 3. Worried about the side effects of B liver drug

treatment

□ 4. Too busy

□ 5. I did not pay for health insurance at their own

expense

□ 6. Asymptomatic unwilling to receive treatment

□ 7. Forgot.

□ 8. Others: Please specify

**Ⅴ、Patient, doctor, resource**

1. Where do you go to see a doctor? [Multiple choice]複選)

□ 1. Health clinic □ 2. Clinic □ 3. Hospital □ 4. Emergency room □ 5. Chinese medicine

□ 6. General pharmacy □ 7. Others, please specify ____________

1. Is there a doctor you visit regularly?

□ 1. Yes □ 2. No

1. In the last three months, have you bought a drug using radio advertising?

□ 1. Yes, including: ________________________ 2. No

**Ⅵ、Hepatitis B, hepatitis C knowledge**

**A、Hepatitis B infection**

**1. Do you think the following is the transmission of hepatitis B? (Check one answer on each line)**

|  | Yes | No | Unknow |
| --- | --- | --- | --- |
| 1. He was born from infected mothers to infants |  |  |  |
| 1. Hepatitis B is suffering from breast-feeding mothers |  |  |  |
| 1. And dine with hepatitis B patients |  |  |  |
| 1. Ate hepatitis B patients prepared food |  |  |  |
| 1. Eat hepatitis B patients chewed food |  |  |  |
| 1. And hepatitis B patients sharing toothbrushes |  |  |  |
| 1. Sharing razor with hepatitis B patients |  |  |  |
| 1. Hepatitis B patients cough or sneeze spray |  |  |  |
| 1. And hepatitis B patients holding hands |  |  |  |
| 1. And hepatitis B patients with sex |  |  |  |

**2. Consequences of hepatitis B virus infection: The following describes you think ...**

|  | Yes | No | Unknow |
| --- | --- | --- | --- |
| 1. Hepatitis B patients with hepatitis B will have a lifetime |  |  |  |
| 1. Not properly track the treatment of hepatitis B can lead to liver cancer and cirrhosis of the liver |  |  |  |
| 1. Hepatitis B is not easy to cure, but the disease can be controlled |  |  |  |
| 1. Most patients with chronic hepatitis B have no symptoms |  |  |  |
| 1. Hepatitis B patients that appear healthy can still transmit B-liver |  |  |  |

**B、Hepatitis C infection**

**1、Do you think the following is the transmission of hepatitis C ? (Check one answer on each line)**

|  | Yes | No | Unknow |
| --- | --- | --- | --- |
| 1. Hepatitis C can be transmitted from mothers to infants infected at birth |  |  |  |
| 1. Is hepatitis C patients to donate blood can lead to hepatitis C infection |  |  |  |
| 1. There are people with hepatitis C can lead to work with hepatitis C infection |  |  |  |
| 1. With the blood of patients with hepatitis C, a needle or sharp objects may cause the bar to Hepatitis C infections |  |  |  |
| 1. injecting illegal drugs can lead to hepatitis C infection |  |  |  |
| 1. Patients handshake with hepatitis C can lead to hepatitis C infection |  |  |  |
| 1. Kiss patients with hepatitis C can lead to hepatitis C infection |  |  |  |
| 8. Sexual behavior with hepatitis C patients can lead to hepatitis C  infection |  |  |  |

**2. Consequences of hepatitis C virus infection: The following describes what you think ...**

|  | Yes | No | Unknow |
| --- | --- | --- | --- |
| 1. If infected with hepatitis C virus infection is likely to be a lifetime |  |  |  |
| 1. Not properly track the treatment of hepatitis C can lead to liver cancer and cirrhosis of the liver |  |  |  |
| 1. Hepatitis C can be cured |  |  |  |
| 1. Most of the patients with hepatitis C do not have symptoms |  |  |  |
| 1. Apparently healthy patients with hepatitis C can still spread hepatitis C |  |  |  |

**Ⅶ、Other factors Have you ever had the following experience? (Check one answer on each line)**

**Personal Data Protection Management:**

|  | Yes | No | Unknow |
| --- | --- | --- | --- |
| 1. Tattoo |  |  |  |
| 2. Transfusion |  |  |  |
| 3. Drug abuse |  |  |  |
| Surgery |  |  |  |
| 5. Shared toothbrush |  |  |  |
| 6. Shared razor |  |  |  |
| 7. Shared needles |  |  |  |
| 8. Acupuncture, cupping |  |  |  |
| 9. Treatment of teeth |  |  |  |

In line with Article 8 of the Personal Data Protection Act, this screening is live To provide you with a more complete medical assessment, diagnosis and advice, will search Set your personally identifiable information, personal and physical description, habits, family circumstances, occupations and health records and other resources. You are free to choose whether to provide, if not provide will not be able to make the correct and favorable treatment for your specific situation. You have the right to request access to, supplement or correct information in accordance with the provisions of Article 3 of the same Act and you may at any time request that the collection or utilization cease.

*Signature / date:*
